# Supplementary material for: Structure-based insights into self-cleavage by a four-way junctional twister-sister ribozyme
Source: Nat Commun. 2017 Oct 30;8:1180. doi: 10.1038/s41467-017-01276-y (PMC5660989; doi:10.1038/s41467-017-01276-y)
Supplement: Supplementary file 1 — Supplementary Information [file 41467_2017_1276_MOESM1_ESM.pdf]

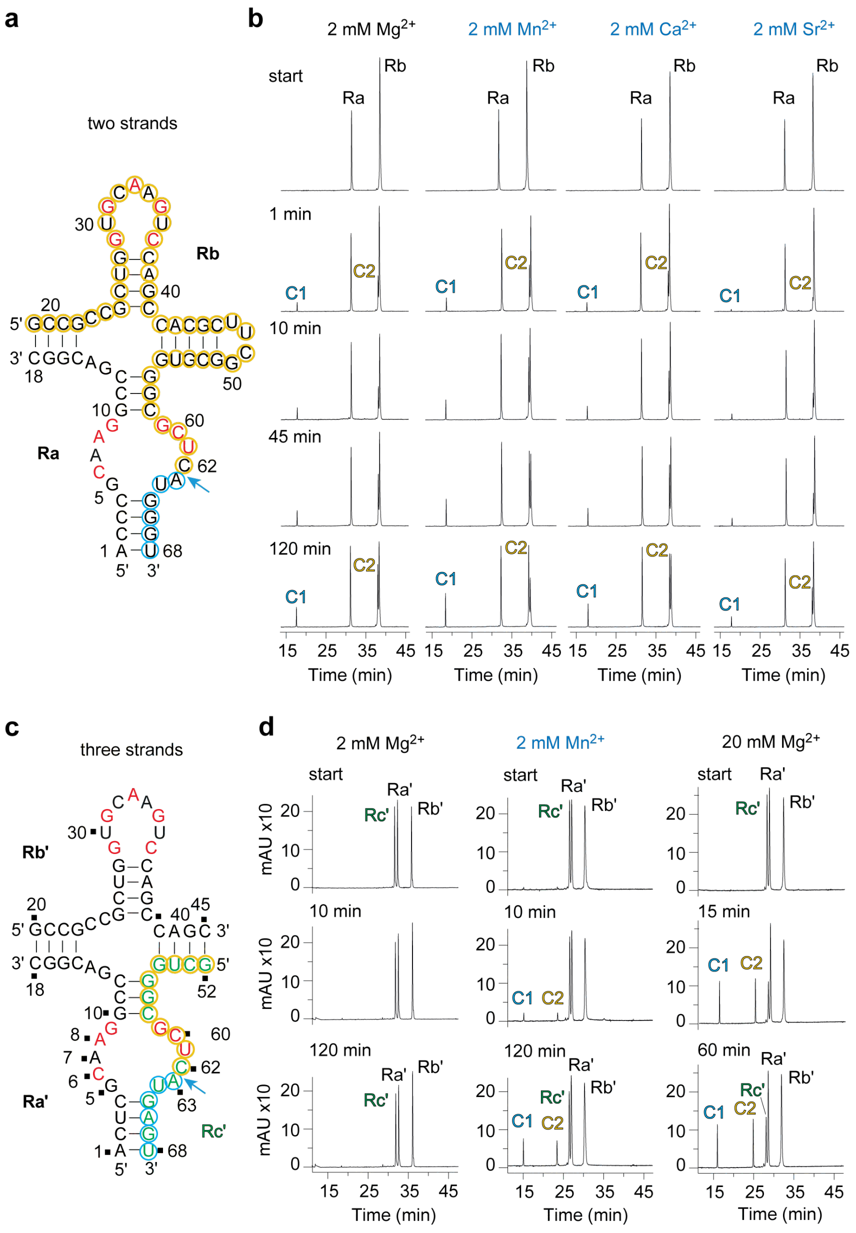

Supplementary Figure 1. Self-cleavage of the Two-stranded and Three-stranded Twister-sister ribozyme.

(a) Two-strand construct of the twister-sister ribozyme (as used for crystallographic studies except dC62C). (b) Cleavage activity of two-stranded ribozyme in the presence of 2 mM Mg^{2+}, 2 mM Mn^{2+}, 2 mM Ca^{2+}, and 2 mM Sr^{2+}.(c) Three-strand construct of the twister-sister ribozyme. (d) Cleavage activity of three-stranded ribozyme in the presence of 2 mM Mg^{2+}, 2 mM Mn^{2+}, and 20 mM Mg^{2+}.

Cleavage activity analyzed at 55 μM RNA each strand; divalent cation concentraion as indicated; 100 mM KCl, 30 mM HEPES, pH 7.5, 23 °C. HPLC conditions: Dionex DNAPac column (4 x 250 mm^2), 80 °C, 1 mL min^{-1}, 0–60% buffer B in 45 min. Buffer A: Tris–HCl (25 mM), urea (6 M), pH 8.0. Buffer B: Tris–HCl (25 mM), urea (6 M), $NaClO_4$ (0.5 M), pH 8.0.

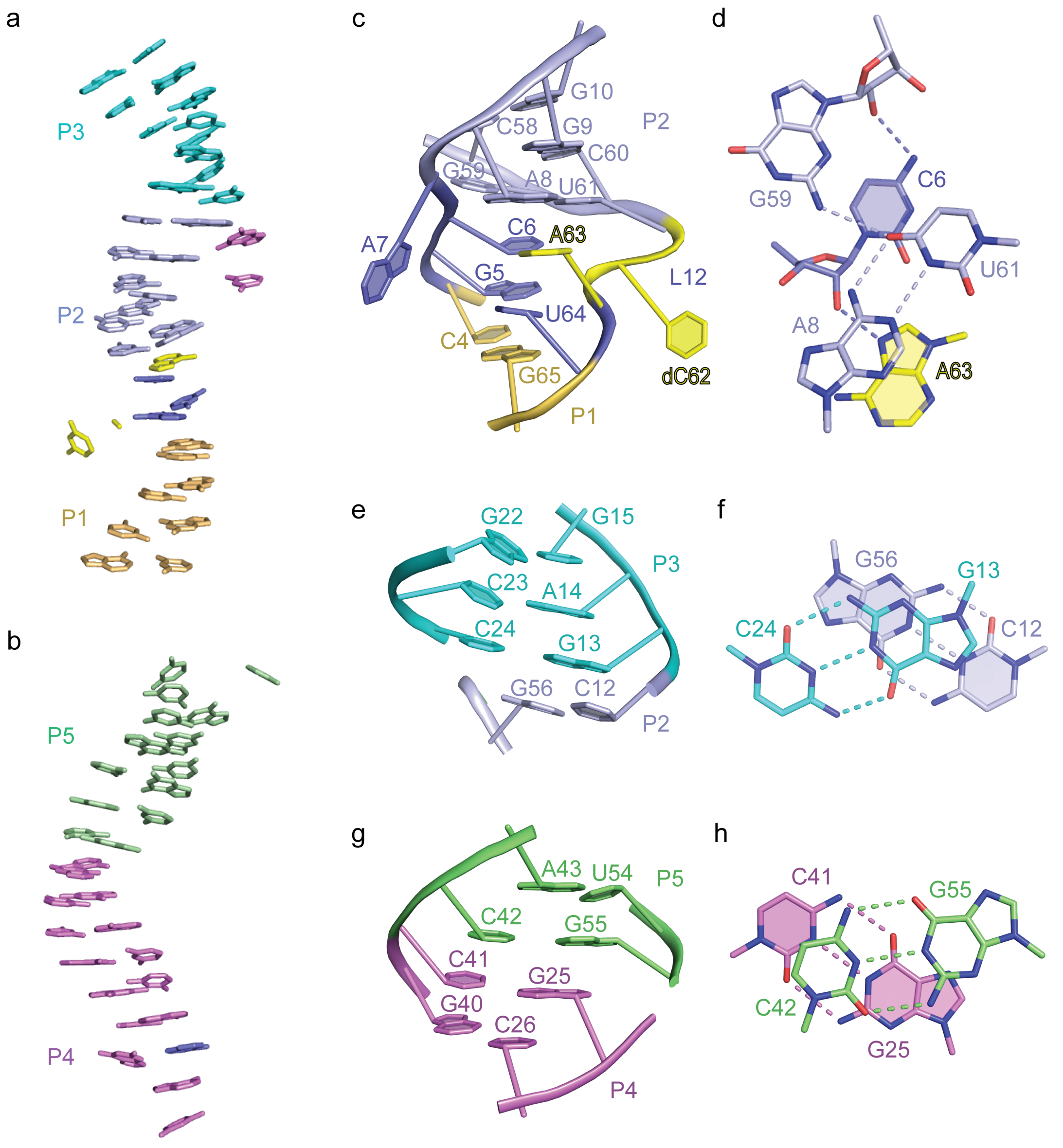

Supplementary Figure 2. Continuous Stacking of Stem Segments in the Tertiary Structure of the Twister-sister Ribozyme.

(a) Continuous stacking of stems P1, P2 and P3 (on proceeding from bottom to top). (b) Continuous stacking of stems P4 and P5 (on proceeding from bottom to top). (c, d) Stacking of adjacent base pairs at the junction of helical stems P1 and P2. Stacking between G59•(A8-U61) base triple and *trans* Hoogsteen-sugar edge A63•C6 pair. (e, f) Stacking of adjacent base pairs at the junction of helical

stems P2 and P3. Stacking between Watson-Crick G56-C12 and C24-G13 base pairs. (g, h) Stacking of adjacent base pairs at the junction of helical stems P4 and P5. Stacking between Watson-Crick G25-C41 and C42-G25 base pairs.

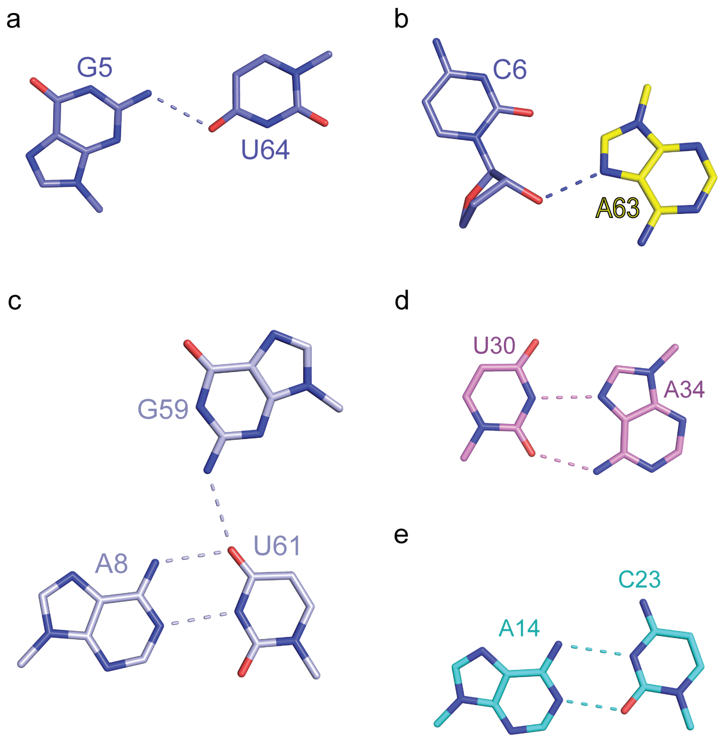

Supplementary Figure 3. Additional Non-canonical Pairing in the Tertiary Structure of the Twister-sister Ribozyme.

(a, b) A non-canonical *trans* G5•U64 pair that extends stem P1 (panel a) and a *trans* sugar edge-Hoogsteen C6•A63 pair formed in loop L1 that extends stem P1 (panel b). (c) Stem P2 was extended by a Watson-Crick A8•U61 pair, which also forms part of a major groove aligned G59•(A8-U61) base triple. (d, e). A *trans* Watson-Crick-Hoogsteen U30•A34 pair formed in SL4 that extends stem P4 (panel d) and a *cis* Watson-Crick-Watson-Crick A14•C23 pair that extends stem P3 (panel e).

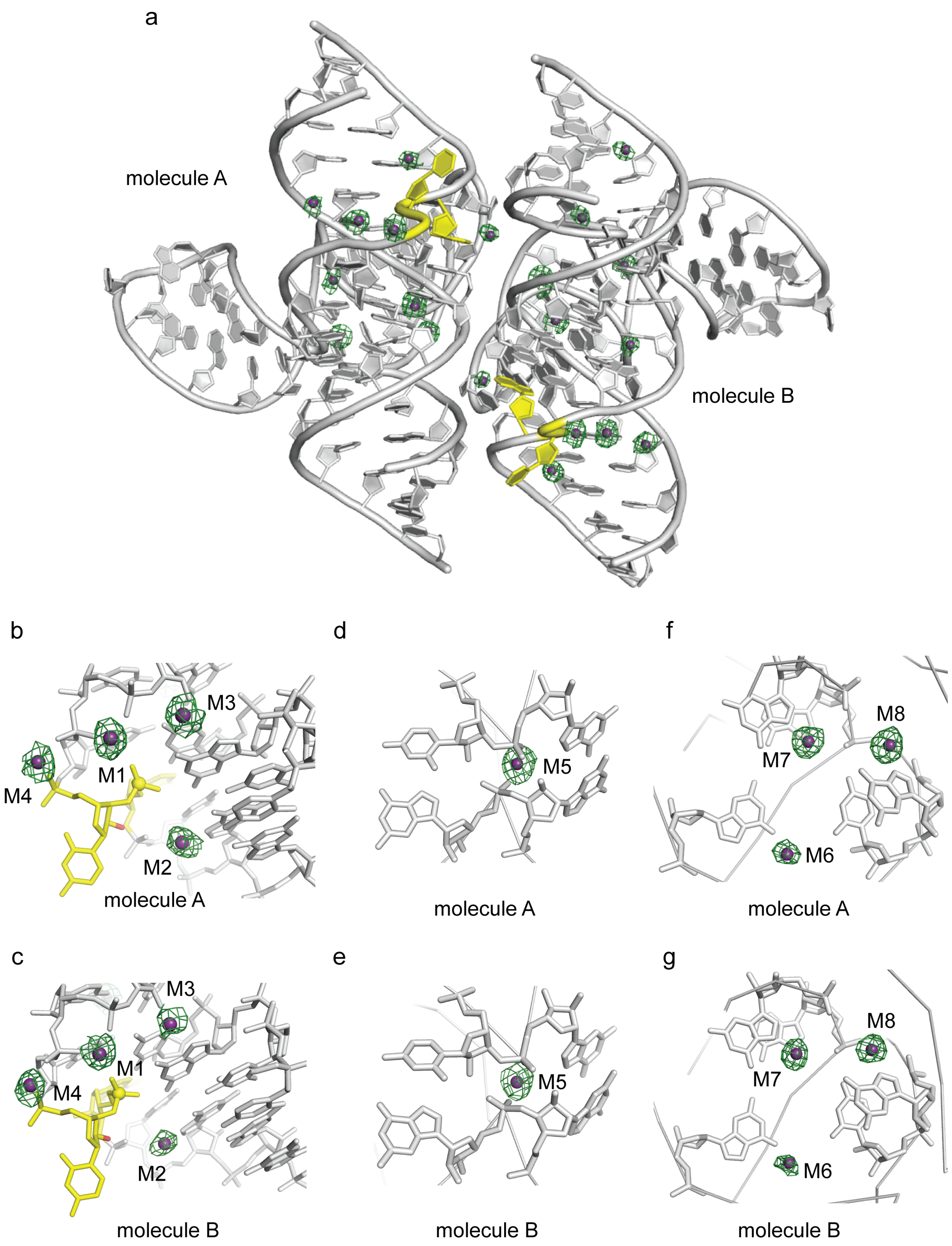

Supplementary Figure 4. Packing Alignments in the Crystal Lattice and Bound Mg^{2+} Sites in Structure of the Twister-sister Ribozyme.

(a) Anomalous map for Mn^{2+} sites (shown as purple balls) involving the two molecules of the four-way junctional twister-sister ribozyme in each asymmetrical

unit. (b, c), Expanded view of panel A for divalent metal ions laeled M1, M2, M3 and M4. (d, e) Expanded view of panel A for divalent metal ion labeled M5. (f, g) Expanded view of panel A for divalent metal ions labeled M6, M7 and M. Anomalous density (3.0 σ level) for the four-way junctional twister-sister ribozyme in 50 mM Mn^{2+} soaked solution in panels a to g.

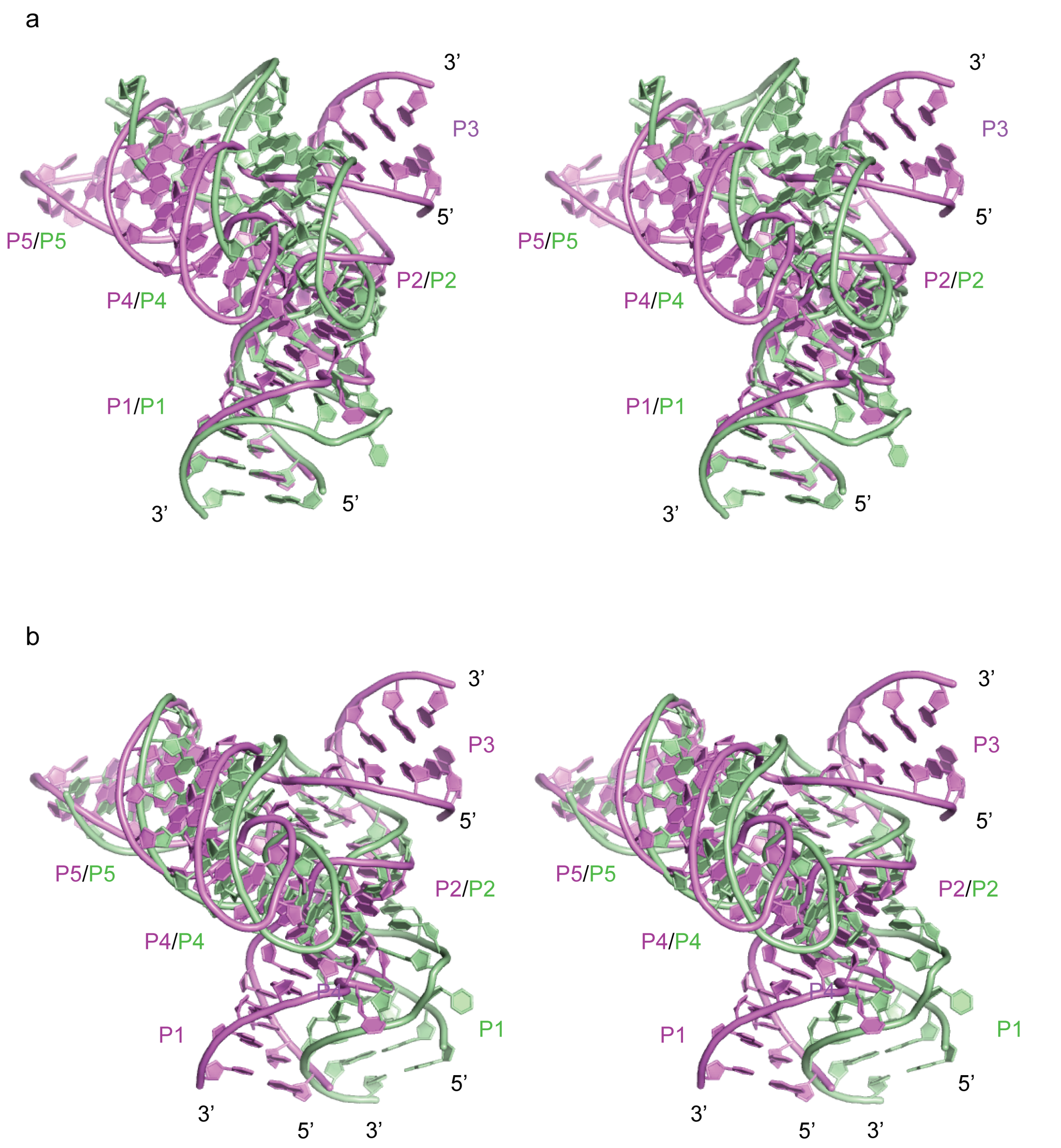

Supplementary Figure 5. Two Alternate Stereo Views Comparing Crystal Structures of the Twister-sister Ribozyme.

(a) Stereo views showing superposition of the crystal structures of the twister-sister ribozyme color-coded in magenta (this study) and green (PDB: 5T5A) with the alignment of Stem P1. (b) An alternate stereo view of the comparison from that shown in panel a with the alignment of Stem P4 and P5.

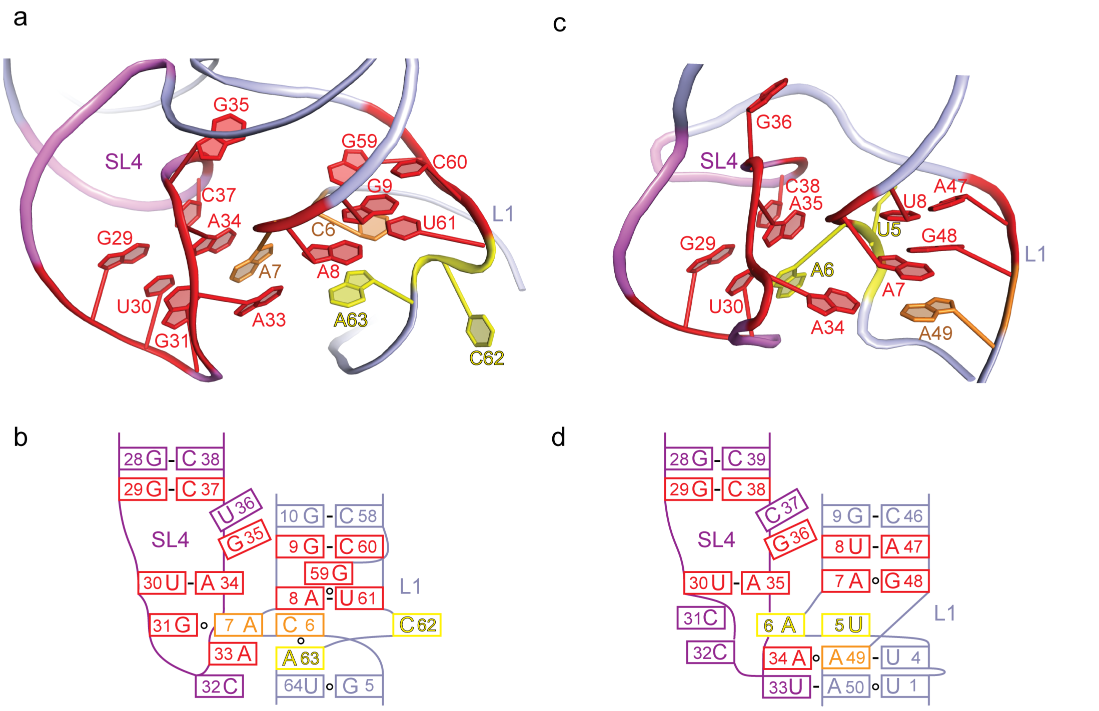

Supplementary Figure 6. Long-range Loop-loop Interactions Bringing Together Conserved Residues in Our Structures of the Twister-sister and Twister Ribozymes.

(a, b) Bringing together of conserved residues (in red) following interaction between the internal and stem loops in the tertiary structure of the four-way junctional twister-sister ribozyme (panel a; this study), together with a schematic of the junctional interactions as shown in panel b. (c, d) Bringing together of conserved residues (in red) following interaction between the internal and stem loops in the tertiary structure of the twister ribozyme (panel c; PDB code: 5DUN), together with a schematic of the junctional interaction as shown in panel d.

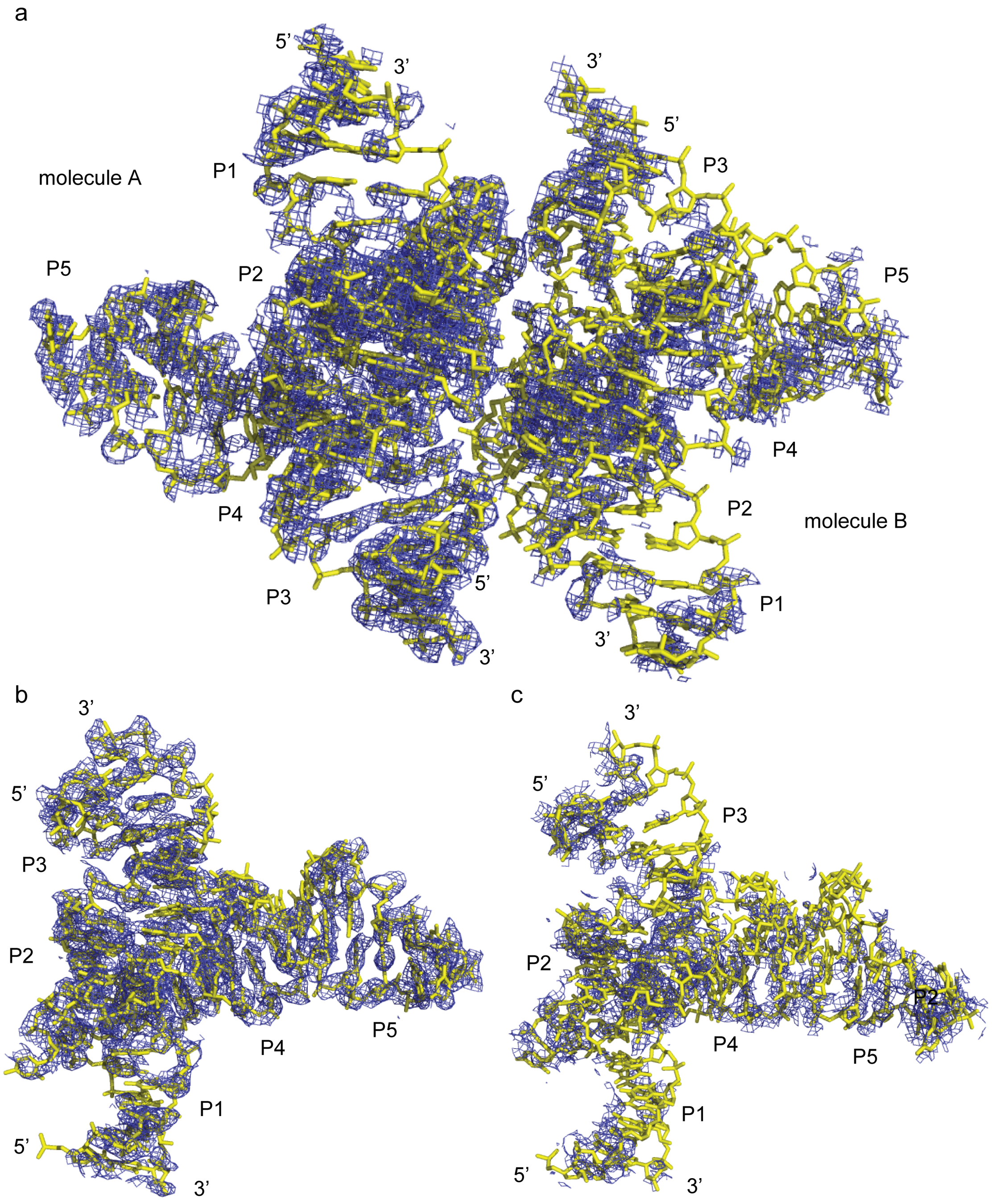

Supplementary Figure 7. Fitting of the Final Twister-sister Ribozyme Structure to the Initial Experimentally-phased Fourier Map.

(a) The view of the experimentally-phased *2Fo-Fc* electron density (1.0 σ level) for one asymmetric unit of the twister-sister ribozyme and fitting of the final

refined structure to the electron density. (b, c) Two alternate views of each molecule in the asymmetric unit Same as panel a.

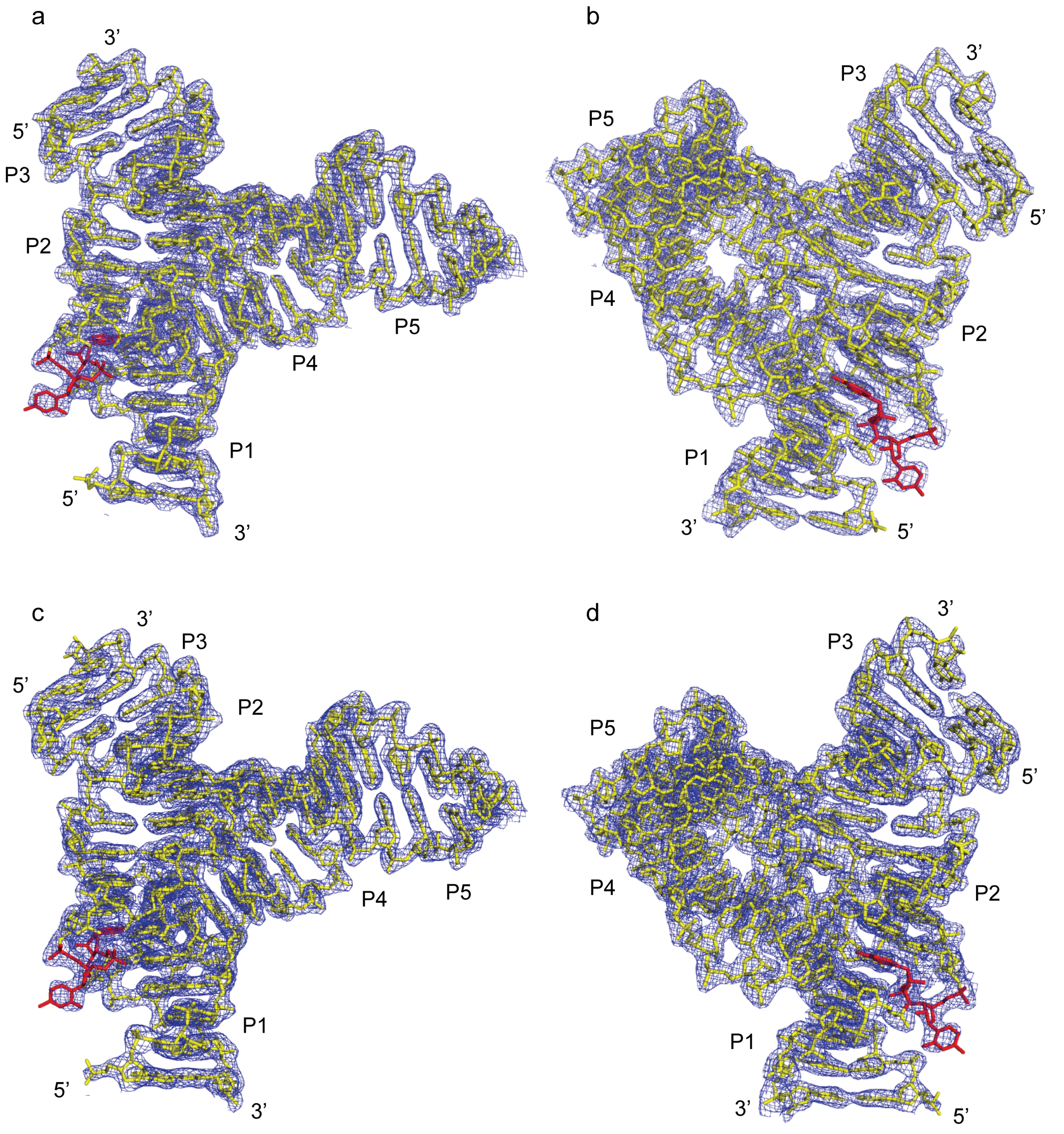

Supplementary Figure 8. Fitting of the Twister-sister Ribozyme Structure to the Electron Density Map.

(a, b) Two alternate views of the 2.0 Å *2Fo-Fc* electron density (1.0 σ level) for one molecule in the asymmetric unit of the twister-sister ribozyme and fitting of the structure to the electron density. (c, d) Same as panel a, b for the second molecule of twister-sister in the asymmetric unit.
